# Toxico-clinical study of patients poisoned with household products; a two-year cross-sectional study

**DOI:** 10.1186/s40360-022-00640-z

**Published:** 2022-12-29

**Authors:** Nastaran Eizadi-Mood, Sahar Sadat Lalehzar, Sara Niknam, Razieh Mahvari, Parisa Mirmoghtadaee, Rokhsareh Meamar

**Affiliations:** 1 Present Address: Department of Clinical Toxicology, School of Medicine, Isfahan Clinical Toxicology Research Center, Isfahan University of Medical Sciences, Isfahan, Iran; 2 Present Address: Isfahan Clinical Toxicology Research Center, Isfahan University of Medical Sciences, Isfahan, Iran; 3 Present Address: Clinical Biochemistry Research Center, Shahrekord University of Medical Sciences, Shahrekord, Iran

**Keywords:** Poisoning, Household products, Corrosive substances, Hydrocarbons, Outcome

## Abstract

**Background:**

Several studies worldwide have investigated household product poisoning. We conducted a toxico-clinical study on the two-year prevalence of poisoning with household products.

**Methods:**

This cross-sectional study was performed in Khorshid Hospital, the main referral center for poisoning cases in Isfahan, affiliated to Isfahan University of Medical Sciences, Isfahan, central Iran. All patients with intentional or unintentional household substance poisoning, referring to the poisoning emergency center of the hospital, were evaluated with respect to epidemiological and toxico-clinical features and outcomes.

**Results:**

During the study period, 5946 patients were hospitalized, of which 83 (1.39%) had been poisoned with household products including 48 (57.8%) men and 35 (42.2%) women with a mean ± SD age of 34.40 ± 17.71 years. Most patients (54.2%) were in the 20–40-year-old age group. Accidental poisoning (63.9%) was the most common type of exposure (*P* = 0.02) predominantly in men (57.8%, *P* = 0.51). The most common household products were sodium hypochlorite (32.53%) followed by petroleum hydrocarbon (21.68%). Most of the accidental poisonings (77.8%) were due to petroleum hydrocarbon. 59% of cases were poisoned at home (*P* < 0.0001). No patient died.

**Conclusion:**

Household products were not common means of poisoning in our referral center. Sodium hypochlorite and petroleum hydrocarbon were the most common substances. Most of the patients were men with accidental exposure at home. Because of the availability of the household product, the frequency and outcomes may be varied in different societies.

## Introduction

Poisoning has been a main global public health matter. The prevalence and types of poisoning differ extensively across the world and depend on socioeconomic status and cultural issues, as well as on local industrial and agricultural accomplishments [1]. The World Health Organization (WHO) Global Burden of Disease project presented that an estimated 345,814 people of all ages deceased worldwide because of accidental poisoning in 2008. Low-income and middle-income countries have greater poisoning death rates than high-income countries [2].

In developing countries, household substances and insecticides are the common causes of poisoning [3]. Acute pediatric poisoning especially with household products remains a worldwide health issue that requires medical attention in hospital emergency departments with consequences such morbidity and mortality [4–6]. In 2018 alone, the US poison control centers were contacted nearly 2.1 million times in relation to suspected human poisonings. In approximately 11% of cases, the poisonings were caused by household cleaning products [7]. A variety of substances including solvents, preservatives, odor agents, and biocidal active ingredients, in common household products are potentially hazardous [8].

Several studies around the world have investigated household product poisoning. In Victoria Hospital, Bangalore, among 266 cases of poisoning, 4.6% of deaths were reported due to corrosive acid consumption, the most common being hydrochloric acid followed by sulfuric acid [9]. The information released by the Poison Control Center of Ain Shams University Hospitals (PCC-ASUH) in Egypt, household chemicals including corrosives and detergents were the most commonly involved toxic substances [10]. Chemicals and alcohol sanitizer poisonings were the highest household substance toxicities in Saudi Arabia. Also, in a study conducted in Italy, 229,040 poisoning cases were attributed to accidental poisoning by cleaning products [11].

Household substance poisoning has social, economic, and health implications especially in children under the age of five who account for the largest percentage of poisonings worldwide [4, 12]. The development of respiratory hypersensitivity or asthmatic symptoms in some susceptible individuals has been associated with the careless use of household products and exposure to specific substances [13].

Demographic variables, house structure, family type, income, the number of siblings, and previous poisoning incidents have been reported as the major determinants in household poisoning [14]. The outcome of poisoning ranges from mild incidences to severe complications or death; most pediatric poisonings occur by accident and oral routes of exposure [4, 12]. As the availability of household products and outcomes may be varied in different societies, we conducted a two-year cross-sectional study on the toxico-clinical features of poisoning with household products in the poisoning referral center of Isfahan province, central Iran.

## Methods

This cross-sectional study was performed in 2020 in Khorshid Hospital affiliated to Isfahan University of Medical Sciences. The study was approved by the Ethics Committee of Isfahan University of Medical Sciences (code: IR.MUI.MED.REC.1397.315). All adult and children with intentional or unintentional household substance poisoning, referring to the poisoning emergency center of Khorshid hospital, the main referral center for poisoning cases in Isfahan province, from 11th January 2015 to 17th October 2016 were included.

Based on recorded data, household products were defined as hydrochloric acid, sodium hypochlorite, detergent/shampoo, combination household products, and others (antiseptics such as chlorhexidine, hydrogen peroxide, iodine and iodophors, potassium permanganate, and chlorine, disinfectants [formaldehyde, phenol]), boric acid, and chlorates).

We excluded patients who were discharged against medical advice and those whose records were incomplete (more than 20%). The medical files of patients were extracted based on ICD-10 codes (Antiseptics, Disinfectants, Sterilant compounds, Camphor, Moth Repellents, Caustics, Hydrofluoric Acid and Fluorides and Hydrocarbons such as naphthalene) for household products. Data were recorded in the data gathering form. Demographic information includes age, sex, occupation, kind of household product, the time interval of poisoning to admission, type of exposure (intentional, accidental, unknown), routes of exposure (ingestion, inhalation, dermal, unknown, more than one route of exposure), history of addiction, type of addiction (alcohol, cigarettes, methadone), medical history related to psychiatric illness, having a history of current medical problems (diabetes mellitus, hypertension, cardiovascular disease, and respiratory disease), and clinical manifestations on admission were included in the data gathering form.

Nausea, vomiting, and abdominal pain were considered manifestations of gastrointestinal involvement. Palpitation, chest pain, and hemodynamic changes (tachycardia, bradycardia, hypotension, and hypertension) were considered abnormal cardiovascular manifestations. Dyspnea, cough, mouth secretions, abnormal lung auscultation were considered abnormal respiratory functions.

Data were analyzed using SPSS 15 software (SPSS Inc. Chicago USA). Results were presented as frequency (percent) or mean (standard deviation) and median. *P* < 0.05 was considered statistically significant. The outcome of the patients was defined as survived without complication, survived with complications (including respiratory, renal, cardiovascular, and neurological complications), and death. Categorical data were compared using Fisher’s exact and Chi-square tests. The means of variables were compared using the two-way repeated-measure ANOVA or independent *t* test. For evaluation of correlation between different variables, a Spearman correlation test was performed.

## Results

During the study period, 87 patients were admitted because of household product poisoning. Four patients were excluded and the data of 83 patients were analyzed.

**Fig. 1:**
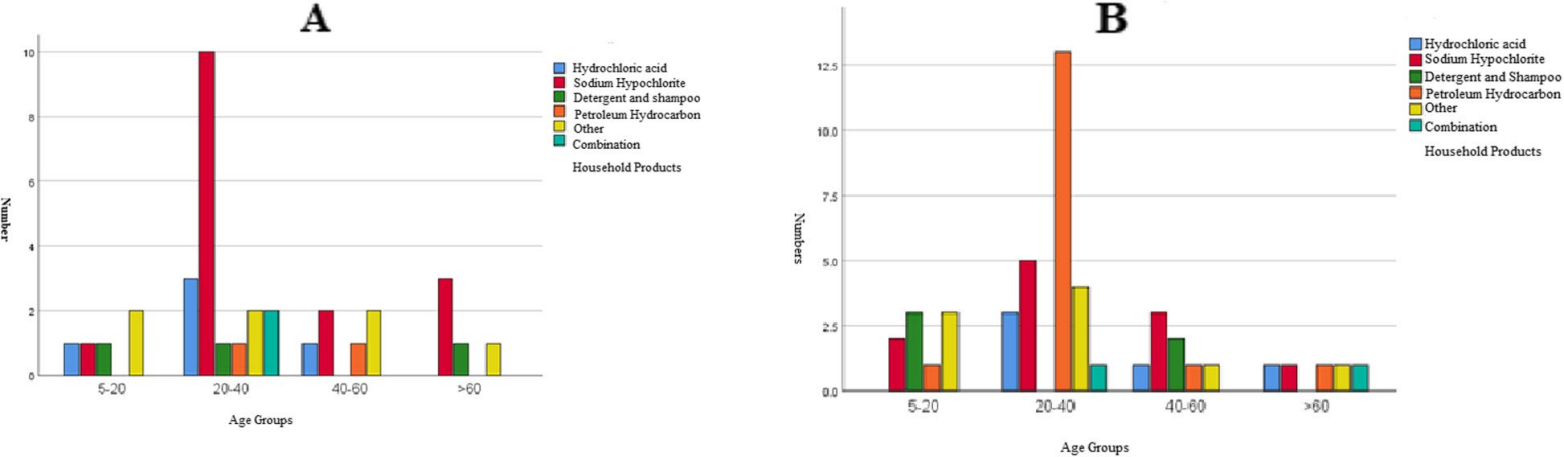
Household products poisoning with respect to gender in different age groups (**A**: female, **B**: male)

The study population consisted of 48 (57.8%) men and 35 (42.2%) women with a mean ± SD age of 34.40 ± 17.71 years. The common household products causing poisoning were sodium hypochlorite (32.53%) followed by petroleum hydrocarbon (21.68%). Initial analysis of demographic, drug toxicity information, and medical history was performed. Intentional poisoning was more common among men (57.8%) compared with women (42.2%), but the difference was not statistically significant (*P* = 0.51, Fig. 1). The mean ± SD ages in women and men were 35.83 ± 18.50 and 33.35 ± 17.24 years, respectively. Table 1 shows the information regarding the epidemiology and toxico-clinical features. Most patients (54.2%) were in the 20–40-year-old age group (Table 2).

**Table 1:** Comparison of different variables in patients with household products poisoning

	household products								
Variables		Hydrochloric acid (*N* = 10)	sodium hypochlorite (*N* = 27)	Detergent /shampoo (N = 8)	Petroleum hydrocarbon (*N* = 18)	Others (*N* = 16)	combination household products (*N* = 4)	Total (*N* = 83)	*P* value
Gender n (%)	Men	5 (50.0)	11 (40.7)	5 (62.5)	16 (88.9)	9 (56.3)	2 (50.0)	48 (57.8)	0,51
	women	5 (50.0)	16 (59.3)	3 (37.5)	2 (11.1)	7 (43.8)	2 (50.0)	35 (42.2)	
Marriage n (%)	Single	5 (50)	11 (40.7)	4 (50)	9 (50)	9 (56.3)	1 (25)	39 (47)	0,88
	Married	5 (50)	16 (59.3)	4 (50)	9 (50)	7 (43.8)	3 (75)	44 (53)	
Nationality n (%)	Iranian	9 (90)	25 (92.6)	8 (100)	17 (94.4)	14 (87.5)	4 (100)	77 (92.8)	0.93
	Other nationality	1 (10)	2 (7.4)	0 (0)	1 (5.6)	2 (12.5)	0 (0)	6 (7.2)	
Age (mean ± SD)		35.70 ± 15.2	35.81 ± 18.27	28.38 ± 22.56	33.50 ± 13.79	33.38 ± 20.19	41.75 ± 18.22	34.40 ± 17.71	0.86
Med (min-max)		32 (8–26)	32 (6–70)	22 (6–63)	31.50 (6–68)	32 (6–78)	35.5 (28–68)	32 (6–78)	
Number of children per family	0	3 (37.5)	12 (57.1)	4 (80)	12 (58.3)	7 (58.3)	1 (50)	39 (61.9)	0.76
	1	2 (25)	3 (14.3)	0 (0)	1 (6.7)	2 (16.7)	0 (0)	8 (12.7)	
	> 1	3 (37.5)	6 (28.6)	1 (20)	2 (13.3)	3 (25)	1 (50)	16 (25.4)	
Type of admission	Transfer from other centers	0 (0)	2 (7.4)	0 (0)	1 (5.6)	2 (12.5)	0 (0)	5 (6)	0.8
	Emergency services	0 (0)	3 (11.1)	0 (0)	0 (0)	2 (12.5)	0 (0)	5 (6)	
	Admission in Hospital	10 (100)	22 (81.5)	8 (100)	17 (94.4)	12 (75)	4 (100)	73 (88)	
Route of exposure	Ingestion	8 (80)	25 (92.6)	8 (100)	18 (100)	14 (87.5)	2 (50)	75 (90.4)	0.25
	inhalation	1 (10)	2 (7.4)	0 (0)	0 (0)	0 (0)	2 (50)	5 (6)	
	Dermal	0 (0)	0 (0)	0 (0)	0 (0)	1 (6.3)	0 (0)	1 (1.2)	
	Combination*	0 (0)	0 (0)	0 (0)	0 (0)	1 (6.3)	0 (0)	1 (1.2)	
	Unknown	1 (10)	0 (0)	0 (0)	0 (0)	0 (0)	0 (0)	1 (1.2)	
Type of poisoning	suicidal	5 (50)	10 (37)	1 (12.5)	1 (5.6)	4 (25)	1 (25)	22 (26.5)	0.02
	Accidental	5 (50)	15 (55.6)	6 (75)	14 (77.8)	11 (68.8)	2 (50)	53 (63.9)	
	Unknown	0 (0)	2 (7.4)	1 (12.5)	3 (16.7)	1 (6.3)	1 (25)	8 (9.6)	
Place of poisoning	Home	6 (60)	21 (77.8)	4 (50)	4 (22.2)	11 (68.8)	3 (75)	49 (59)	0.00
	Work place	4 (40)	3 (11.1)	1 (12.5)	10 (55.6)	0 (0)	0 (0)	18 (21.7)	
	Other places	0 (0)	0 (0)	0 (0)	0 (0)	3 (18.8)	0 (0)	3 (3.6)	
	Unknown	0 (0)	3 (11.1)	3 (37.5)	4 (22.2)	2 (12.5)	1 (25)	13 (15.7)	
History of Addiction	Yes	0 (0)	5 (18.5)	0 (0)	2 (11.1)	2 (12.5)	0 (0)	9 (10.8)	0.74
kind of addiction	Opium	0 (0)	1 (3.7)	0 (0)	0 (0)	0 (0)	0 (0)	1 (1.2)	0.99
	Heroin	0 (0)	1 (3.7)	0 (0)	0 (0)	0 (0)	0 (0)	1 (1.2)	
	Cigarette	0 (0)	1 (3.7)	0 (0)	0 (0)	2 (12.5)	0 (0)	3 (3.6)	
	other	0 (0)	1 (3.7)	0 (0)	0 (0)	0 (0)	0 (0)	1 (1.2)	
	combination	0 (0)	1 (3.7)	1 (5.6)	1 (5.6)	0 (0)	0 (0)	2 (2.4)	
	Not mentioned	10 (100)	22 (81.5)	8 (100)	17 (94.4)	14 (87,5)	4 (100)	75 (90.4)	
history of psychological problems	Yes	2 (20)	1 (3.7)	1 (12.5)	0 (0)	3 (18.8)	0 (0)	7 (8.4)	0.27
Under the treatment of psychiatrist	Yes	2 (20)	1 (3.7)	0 (0)	0 (0)	2 (12.5)	0 (0)	5 (6)	0.23
History of Suicide	Yes	1 (10)	1 (3.7)	0 (0)	0 (0)	1 (6.3)	0 (0)	3 (3.6)	0.73
history of suicide in Family	Yes	0 (0)	0 (0)	1 (12.5)	0 (0)	1 (6.3)	0 (0)	2 (2.4)	0.21
History of medical disorder	Yes	2 (20)	5 (18.5)	3 (37.5)	2 (11.1)	4 (25)	1 (25)	17 (20.5)	0.69
History of Drugs	Yes	4 (40)	5 (18.5)	1 (12.5)	2 (11.1)	6 (37.5)	1 (25)	19 (22.9)	0.31
Time from exposure to hospital admission (hours)		3.1 ± 4.8	2.4 ± 6.07	2.5 ± 2.2	2.11 ± 2.2	1.3 ± 1.18	1.7 ± 1.1	2.22 ± 4.01	0.95
Mean ± SD (median, minimum-maximum)		(2, .10–13.74)	(1, 0–30)	(2, 0.5–6.75)	(1.35,0.5–8.50)	(1, 0.5–4)	(2, 0.5–2.70)	(1, 0–30)	

Chi square/fisher exact test was used for analysis. *P* value < 0.05 was considered as significant; * more than one route of exposure; A combination household product has been considered two or more than one product (Hydrochloric acid, sodium hypochlorite Detergent /shampoo, Petroleum hydrocarbon)

**Table 2:** Comparison of different household products poisoning with respect to age groups

		Hydrochloric acid (*N* = 10)	sodium hypochlorite (*N* = 27)	Detergent and shampoo (*N* = 8)	Petroleum hydrocarbon (*N* = 18)	Other (*N* = 16)	combination household products (*N* = 4)	Total (*N* = 83)
age groups (years)	5–20	1	3	4	1	5	0	14
		10.0%	11.1%	50.0%	5.6%	31.3%	0.0%	16.9%
	20–40	6	15	1	14	6	3	45
		60.0%	55.6%	12.5%	77.8%	37.5%	75.0%	54.2%
	40–60	2	5	2	2	3	0	14
		20.0%	18.5%	25.0%	11.1%	18.8%	0.0%	16.9%
	> 60	1	4	1	1	2	1	10
		10.0%	14.8%	12.5%	5.6%	12.5%	25.0%	12.0%

*P* value > 0.05; Fisher exact test was used for statistical analysis; A combination household product has been considered two or more than one product (Hydrochloric acid, sodium hypochlorite Detergent /shampoo, Petroleum hydrocarbon)

**Fig. 2:**
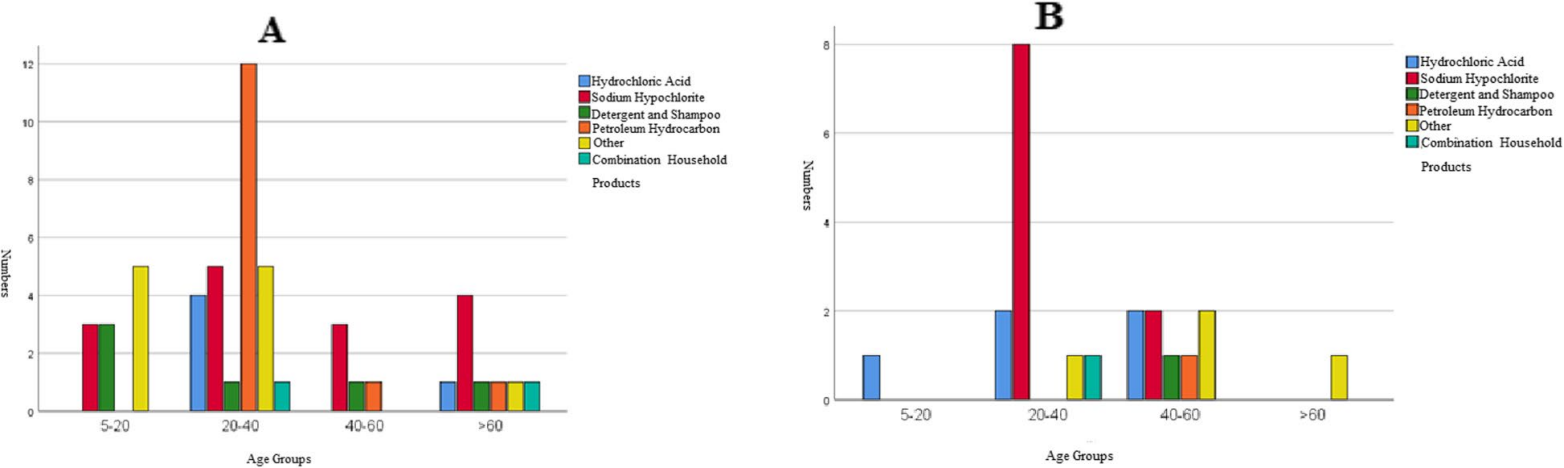
Household products poisoning with respect to route of exposures in different age groups (**A**: accidental, **B**: intentional)

Accidental poisoning was the most common type of exposure (63.9%, *P* = 0.02) and was observed more with hydrochloric acid sodium hypochlorite (Fig. 2). The mean ± SD ages of the patients poisoned through the accidental and intentional type of exposure were 34.75 ± 19.57 and 35.50 ± 13.83 years, respectively. Household products poisoning with respect to route of exposures in different age groups has been shown in Fig. 2.

**Fig. 3:**
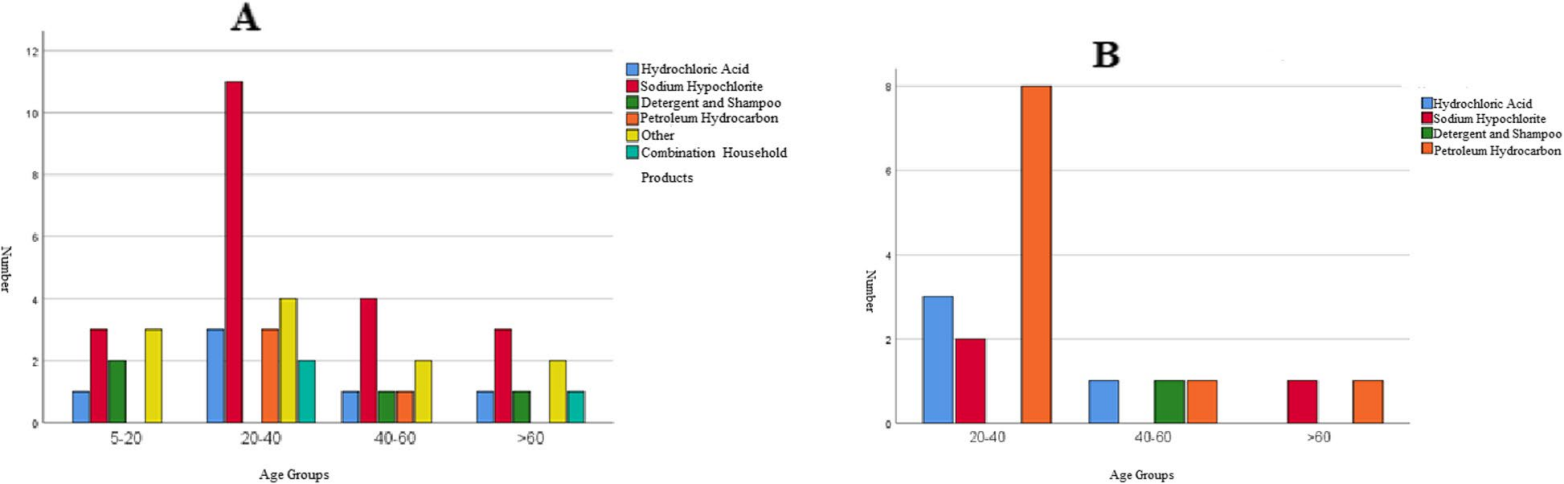
Household products poisoning with respect to place of poisoning in different age groups (**A**: poisoning at home, **B**: poisoning at work)

According to the place of poisoning, most of the patients aged 20–40 years were poisoned by sodium hydrochloride at home, and patients aged 20–40 years were poisoned by petroleum hydrocarbon at work (Fig. 3). The mean ± SD ages of the patients poisoned at home or at work were 34.73 ± 19.61 and 38 ± 14.14 years, respectively.

Most accidental poisonings (77.8%) were caused by petroleum hydrocarbon. 59% of the patients were poisoned at home (*P* = 0.02), mostly with sodium hypochlorite.

We compared the clinical manifestations of the patients, which are presented in Table 3. There was no significant difference in clinical manifestations with respect to different household products. All toxico-clinical variables were compared in patients with respect to outcome. Also, no patient died.

**Table 3:** Clinical manifestations and outcomes of patients with household products poisoning

			Hydrochloric acid (*N* = 10)	sodium hypochlorite (*N* = 27)	Detergent and shampoo (*N* = 8)	Petroleum hydrocarbon (*N* = 18)	Other (*N* = 16)	combination household products (*N* = 4)	Total (*N* = 83)	*p*-value
Clinical (manifestations based on organs involvement	Skin	Normal	10 (100)	26 (96.3)	8 (100)	17 (94.4)	15 (93.8)	4 (100)	80 (96.4)	> 0.05
		Dry/ hot/ flashing	0 (0)	1 (3.7)	0 (0)	1 (5.6)	1 (6.3)	0 (0)	3 (3.6)	
	CNS	Alert	10 (100)	26 (96.3)	7 (87.5)	17 (94.4)	15 (93.8)	4 (100)	79 (95.2)	0.83
		Lethargic Obtundation	0 (0)	1 (3.7)	1 (12.5)	1 (5.6)	1 (6.3)	0 (0)	4 (4.8)	
	CV	abnormal	0 (0)	2 (7.4)	1 (12.5)	0 (0)	3 (18.8)	0 (0)	6 (7.2)	0.30
	RS	abnormal	0 (0)	1 (3.7)	0 (0)	1 (5.6)	0 (0)	0 (0)	2 (2.4)	> 0.05
	GI	abnormal	0 (0)	0 (0)	0 (0)	1 (5.6)	0 (0)	0 (0)	1 (1.2)	0.67
Outcome	without complication		7 (70)	24 (88.9)	6 (75)	16 (88.9)	13 (81.25)	4 (100)	70 (84.30)	0.44
	complication		3 (30)	3 (11.1)	2 (25)	2 (11.1)	3 (18.8)	0 (0)	13 (15.7)	

*CV* cardiovascular system, *GI* Gastrointestinal system, *RS* respiratory system, *CNS* Central Nervous System, A combination household product has been considered two or more than one product (Hydrochloric acid, sodium hypochlorite Detergent /shampoo, Petroleum hydrocarbon); Chi square/fisher exact test was used for analysis. *P* value < 0.05 as significant

There was not any correlation between the kind of household product poisoning and different studied variables. There was correlation between the type of poisoning and the place of poisoning (*P* = 0. 00, *r* = 0.48). As none of the patients died, we could not do regression analysis with respect to outcome.

## Discussion

In this study, we evaluated toxico-clinical and epidemiological characteristics of patients with household product poisoning for the first time in the central part of Iran. During the study period, 5946 patients were hospitalized of which 83 were poisoned by household products (ratio: 1.39%). In a study by Malaysia National Poison Centre during a 10-year period, household products (20.1%) were one of the common agents implicated for intentional exposure [15]. The reason for the difference may be related to different types of household products, as we did not include pesticides poisoning in our study. Also, in a retrospective study on patients admitted to the Poison Control Centre of Ain-Shams University Hospital from January to December 2016, the number of patients with acute household product poisoning was 846 [10]. According to a published study in Turkey, household products accounted for 47.0% including main pyrethroids, parade/thermometer mercury, rodenticides, phenyl, detergents, and corrosives [16]. In another retrospective analysis, household chemical poisoning accounted for 20% of all poisoning cases [17]. In another study in Tabriz, Iran, petroleum products (10.8%) and household detergents (9.8%) were considered of all poisoning cases [18]. The different results could be attributed to the fact that acute pediatric poisoning was considered in the mentioned study, while in our survey, all age groups were included. It seems that the higher prevalence of ingestion in this age group was related to their attitude in putting small foreign objects into their mouth.

The most common household products in our study were sodium hypochlorite (32.53%) followed by petroleum hydrocarbon (21.68%). It should be noted that most of the household sodium hypochlorite concentrations ranged between 3 and 5% and higher concentrations (20%) which is generally used in industrial manufacturing are extremely toxic. However, other researchers found that paraffin (Kerosene) was the most common poisoning agent accounting for 68% of the cases [17]. Different availability and use of household products in various regions may be the reason.

Our results showed that all patients were older than 5 years and most patients were in the 20–40-year-old group. Also, in other studies, household product poisoning was most common in children [10, 16, 17]. Kumar and colleagues presented that most cases with household products were less than 5 years old [17]. Also, in the other study, the age groups involved ranged from 1 year to 18 years with a mean age of 10.22 ± 6.83 years [10]. Our referral center is mainly for adult poisoning cases, and children are admitted to other hospitals because of mostly unintentional poisoning.

In our study, most of the poisoning was due to accidental cases (63.9%), although suicide was the type of exposure in 26.9% of the patients. Similarly, most cases were due to accidental poisoning of caustic/corrosive substances (78.1% of all poisonings) in one study on children (73.3%) [16]. Also, most cases were accidental (74%) in another retrospective study of acute pediatric intoxication by household products presented to the Poison Control Center of Ain-Shams University [10]. However, Kumar and colleagues reported that 19% of the patients poisoned by chemical households in Zimbabwe were suicidal [17].

The most common route of intoxication was ingestion (90.4%) and most poisonings happened at home (59%). In a similar study, the most common route of poisoning with caustic/corrosive substances was ingestion (89.4%), and most were ingested inside the house (93.3%) [17].

In our study, the mean time between exposures to hospital admission was 2 hours. However, some patients were admitted to the hospital within 30 hours after exposure. In another study, about half of all poisoned patients (50.9%) were admitted to the emergency department within the first 2 h of ingestion as well [16]. Gastric lavage was performed only in two cases in our study, however, in the mentioned study, gastric lavage was performed on about half of the poisoned children (48.7%) [16]. As most of our patients were young and were accidentally exposed, gastric lavage was not necessary for the patients.

There was no significant relationship between demographic factors and toxico-clinical factors and the kind of household products in our study, which may be attributed to the small number of patients in different types of household product subgroups. 81.9% of our patients recovered without any complications and no death was reported. Household cleaning products have an unpleasant taste and therefore only small amounts may be ingested. This, along with the short time between ingestion and presentation, might have contributed to the fact that there were no fatal outcomes in these cases.

In our study, complications were reported in 18.2% that resolved with supportive treatment. However, in one study, mortality was recorded for 13% of the patients and most of the deaths were suicides [17]. Andýran and colleagues reported an overall mortality rate of 0.4% during the six-year study period [16]. In a retrospective study, 96.7% of the patients with household products poisoning improved while 3.3% died [10]. Also, in another study, two-thirds of the patients with Blackstone poisoning died. Blackstone is a dyeing agent commonly used for hair and contains a strongly toxic compound known as Paraphenylenediamine (PPD) which can cause multi-organ damage and even early and aggressive management is associated with poor outcomes [19]. Different availability of the household product with respect to their inherent toxicity, the ingested amount by the patients, the time between ingestion to hospital admission, and the supportive care are important factors are important factors affecting the survival of patients with household products.

In conclusion, the prevalence of household products during the study period was 1.39%. Most patients were men, aged five to 70 years old. Accidental poisoning was the most common type of exposure. Sodium hypochlorite and petroleum hydrocarbon were the most common substances that occurred mostly at home. All patients survived.

Our study had some limitations. The study was cross-sectional and both adults and children were evaluated in this study. As the outcome and severity of poisoning can be different in these two age groups, a new research specifically in children is suggested. Also, the number of patients in some poisoning was low; therefore, comparison among the different household products may not be applicable. Finally, it was also a single referral center study and may not be generalizable to different settings.

## Data Availability

The datasets generated and/or analyzed during the current study are not publicly available because of the nature of the participants who attempted suicide but are available from the corresponding author on reasonable request.
